# Supplementary material for: Chromothripsis during telomere crisis is independent of NHEJ, and consistent with a replicative origin
Source: Genome Res. 2019 May;29(5):737–49. doi: 10.1101/gr.240705.118 (PMC6499312; doi:10.1101/gr.240705.118)
Supplement: Supplemental Material [file supp_gr.240705.118_Supplemental_file_1.zip › contigs/annotated_contigs/DB57/contig.2.DB57_length_569_mean_cov_6.82601054482.docx]

**DB57_length_569_mean_cov_6.82601054482**

```
TAATGCCCATTTAAAATTGAATAGTGGCTATCCCAATGTTTAATTTTTGTATAGATAAAATTGTTGTTAAATTAGTTCTTTTTGACATA
 >chr7:126406239-126406535 - E=2e-166
ATTAGATATCAGGATACATTTCTTTGTATGCTCCTTTTTAGTTTGGGATAGTAAAACCACGTTATCGGTGTGGAGGAAGATTCTGCTTC

CAGTTTATAGGAGCAGAAGGTGAAGTAGCCAGAGCACTGGAAGCTCATCAGACAGTTGGTACAAAAATAAAGAGAACTGTTTGTTTCTT

AAAATTTAATCTTTCTCACATTGTAACC|A|ACTGAATATTTAGAGGCATCTTAAACCGAGATCATCAGCTTTTTATCTTAAAGTACAC
                             >chr7:132674585-132674824 + E=4e-129
ATCAAATGTCATAATATTCAATATACGGGTGAATAATGGTGGGAATGGGGAGGTAAAGACACCACCATTAAACTGTGTCTTATCAGCAG

GGCTTAATGGCCAATGTGGTGAAACCCCAACTCTACTAAAAATACAAAAATTAGCCGGGCGTGGTGGCGGGTGCCTGTAATCCCAGCTA

CT|CAGGAGGCTAAGGCAGGGGAATCGCTTGAACCTGG
```
